# Pathogenic potential and the role of clones and plasmids in beta-lactamase-producing *E. coli* from chicken faeces in Vietnam

**DOI:** 10.1186/s12917-019-1849-1

**Published:** 2019-04-04

**Authors:** Passoret Vounba, Julie Arsenault, Rianatou Bada-Alambédji, John Morris Fairbrother

**Affiliations:** 1 Department of Pathology and Microbiology, Faculty of Veterinary Medicine, Université de Montréal, the Swine and Poultry Infectious Diseases Research Centre (CRIPA) and the Research Group on Zoonoses and Public Health (GREZOSP), St-Hyacinthe, Quebec Canada; 2 Department of Public Health and Environment, Ecole Inter-Etats des Sciences et Médecine Vétérinaires (EISMV), de Dakar, Senegal; 3 OIE Reference Laboratory for Escherichia coli (EcL), Université de Montréal, 3200 Sicotte, Saint-Hyacinthe, QC J2S 2M2 Canada

**Keywords:** Avian fecal *Escherichia coli*, Antimicrobial resistance, Virulence, Clusters, Plasmids, Vietnam

## Abstract

**Background:**

Antimicrobial resistance (AMR) in food-producing animals is a global public health issue. This study investigated AMR and virulence profiles of *E. coli* isolated from healthy chickens in Vietnam. *E. coli* were isolated from fecal samples collected in five chicken farms located in the provinces of Hoa Binh, Thai Nguyen and Bac Giang in the North of Vietnam. These isolates were examined by disk diffusion for their AMR, PCR for virulence and AMR genes, pulsed-field gel electrophoresis for relatedness between *bla*_*CMY-2*_/*bla*_*CTX-M*_–positive isolates, electroporation for transferability of *bla*_*CMY-2*_ or *bla*_*CTX-M*_ genes and sequencing for mutations responsible for ciprofloxacin resistance.

**Results:**

Up to 99% of indicator isolates were multidrug resistant. Resistance to third-generation cephalosporins (3GC) was encoded by both *bla*_*CTX-M*_ and *bla*_*CMY-2*_ genes; *bla*_*CTX-M*_ genes being of genotypes *bla*_*CTX-M-1*, *− 14*, *− 15*, *− 17*, *− 57*_ and _*− 87*_, whereas ciprofloxacin resistance was due to mutations in the *gyrA* and *parC* genes. Some isolates originating from farms located in different provinces of Vietnam were found to be closely related, suggesting they may have been disseminated from a same source of contamination. Plasmids may also have played a role in the diffusion of 3GC-resistance as the *bla*_*CMY-2*_ gene was located on plasmids A/C and I1, and the *bla*_*CTX-M*_ gene variants were carried by I1, FIB, R and HI1. Plasmids carrying the *bla*_*CMY-2*_/*bla*_*CTX-M*_ genes also co-transferred resistance to other antimicrobials. In addition, isolates potentially capable of infecting humans, of which some produced *bla*_*CMY-2*_/*bla*_*CTX-M*_, were identified in this study.

**Conclusions:**

Both clones and plasmids could be involved in the dissemination of 3GC-resistant *E. coli* within and between chicken farms in Vietnam. These results demonstrate the necessity to monitor AMR and control antimicrobial use in poultry in Vietnam.

**Electronic supplementary material:**

The online version of this article (10.1186/s12917-019-1849-1) contains supplementary material, which is available to authorized users.

## Background

The use of antimicrobials for therapeutic, prophylactic or growth promoter purposes has greatly contributed to improved animal health, welfare and productivity. However, use of antimicrobials is likely to promote antimicrobial resistance (AMR). *E. coli* is known as a bacterial species in which selection for resistance following the use of antimicrobials appears rapidly [1]. In poultry, the level of AMR is further accentuated by the oral administration of drugs. AMR was first observed for the oldest antibiotics that have been used the longest in human or veterinary medicine and which are now recording very high levels of resistance [2]. This resistance now extends to drugs critically important in humans such as third-generation cephalosporins (i.e. ceftriaxone in human health or ceftiofur in veterinary medicine) or fluoroquinolones, severely limiting therapeutic options. The production of Extended-spectrum β-lactamases (ESBLs) or class C β-lactamases (AmpC) is the most important mechanism of *E. coli* resistance against cephalosporins. With more than 600 variants [3], ESBLs/AmpC are associated with resistance to a wide range of antibiotics including fluoroquinolones, trimethoprim-sulfamethoxazole and tetracyclines [4]. In poultry, CTX-M-14 and CMY-2 ESBLs were first reported in Spain [5] before their detection worldwide in chicken farms [6–9]. However, CTX-M-15 is, to date, the most widely distributed ESBL in *E. coli* worldwide [10]. Genes encoding these enzymes are located on transferable genetic elements such as plasmids which may facilitate their spread to other pathogenic enterobacteria. Important incompatibility groups include I1, N, A/C and P/F, and I1 [6, 11].

Avian Pathogenic *E. coli* (APEC), a subset of Extraintestinal pathogenic *E. coli* (ExPEC), are responsible for substantial economical losses in the poultry industry worldwide [12]. The gastro-intestinal tract of apparently healthy chickens can also act as reservoir for ExPEC potentially pathogenic for humans [13, 14]. Therefore, the development of AMR in the intestinal microflora of chickens could be a source of human contamination by multi drug resistant (MDR) pathogens.

In Vietnam, many classes of antimicrobials, including those of critical importance in human health, are used in high amounts in poultry, often without veterinary prescription [15, 16]. This extensive use of antimicrobials could explain the very high levels of resistance reported for *E. coli* isolated from poultry in this country in several studies [17–19]. However, the role of clones and plasmids in the spread of this AMR is not yet elucidated. The aim of this study was to characterize *E. coli* isolates from chicken faecal samples collected in farms in Vietnam with regard to their virulence and AMR in order to elucidate the role of clones and replicon plasmids in spreading of AMR between and within farms.

## Results

### Isolate collections

In the indicator collection, four morphologically different isolates were obtained from each sample except one from which only three lactose-*uidA* positive isolates were obtained. Thus, this selection method yielded a total of 203 indicator isolates recovered from the 51 samples. In the potential ExPEC collection, 48 isolates, originating from 30 samples from the five farms, were positive for at least one of the tested virulence genes; the number of isolates per sample ranged from 1 to 3 with a median of 1. In the potential ESBL/AmpC collection, a maximum of five isolates morphologically different per sample, when available, were selected, giving a total of 126 potential ESBL/AmpC isolates originating from 31 samples; the number of isolates per sample ranged from 1 to 5 with a median of 5.

### Prevalence of antimicrobial resistance

In order for the presentation of our results to be consistent with the definitions proposed in the literature by an expert panel [20, 21], resistant and intermediate isolates were grouped together (nonsusceptible isolates) to define multidrug resistance. At the isolate level, the highest prevalence of nonsusceptibility was observed in tetracycline (97.5%), ampicillin (95.6%), sulfisoxazole (94.6%) and trimethoprim-sulfamethoxazole (94.1%) and the lowest prevalence was in ceftriaxone, ceftiofur and cefoxitin (3.9% each) and amoxicillin-clavulanic acid (3.4%) (Table 1). Prevalence of nonsusceptibility was also much higher for antimicrobials belonging to the class of aminoglycosides (54.2 to 68.5%) and ciprofloxacine (59.6%). All farms carried at least one indicator isolate nonsusceptible for each of the antimicrobials tested, except for ceftriaxone, ceftiofur and cefoxitin (each 80.0% of farms) and amoxicillin-clavulanic acid (60.0% of farms) (Table 1).

**Table 1:** Prevalence of antimicrobial resistance at isolate and farm level of indicator *Escherichia coli* isolated from healthy chickens in Vietnam

Unit of study (No. examined)	Percentage (%) of units with one or more nonsusceptible isolates per category^a^, antimicrobial class^b^ and antimicrobial^c^													
	Critically important										Highly important			
	Highest priority				High priority									
	FLQ		CPS		PEN	PEN/I	AMG			CPM	FOL		PHE	TET
	NAL	CIP	TIO	CRO	AMP	AMC	GEN	KAN	STR	FOX	SXT	SSS	CHL	TET
Isolates (*n* = 203)	83.7	59.6	3.9	3.9	95.6	3.4	54.2	65.5	68.5	3.9	94.1	94.6	86.2	97.5
Farms (*n* = 5)	100	100	80.0	80.0	100	60.0	100	100	100	80.0	100	100	100	100

^a^Category of human antimicrobial importance according to the World Health Organization (WHO) [66]

^b^Antimicrobial classes: *FLQ* Fluoroquinolones, *PEN/I* Penicillin+β-Lactamase inhibitors, *CPS* Cephalosporines, *AMG* Aminoglycosides, *CPM* Cephamycin, *PEN* Penicillin, *FOL* Folate inhibitors, *PHE* Phenicols, *TET* Tetracyclines

^c^Antimicrobials: *NAL* Nalidixic acid, *CIP* Ciprofloxacin, *AMC* Amoxicillin/clavulanic acid, *TIO* Ceftiofur, *CRO* Ceftriaxone, *AMP* Ampicillin, *FOX* Cefoxitin, *GEN* Gentamicin, *KAN* Kanamycin, *STR* Streptomycin, *SXT* Trimethoprim-sulphamethoxazole, *SSS* Sulfisoxazole, *CHL* Chloramphenicol, *TET* Tetracycline

**Fig. 1:**
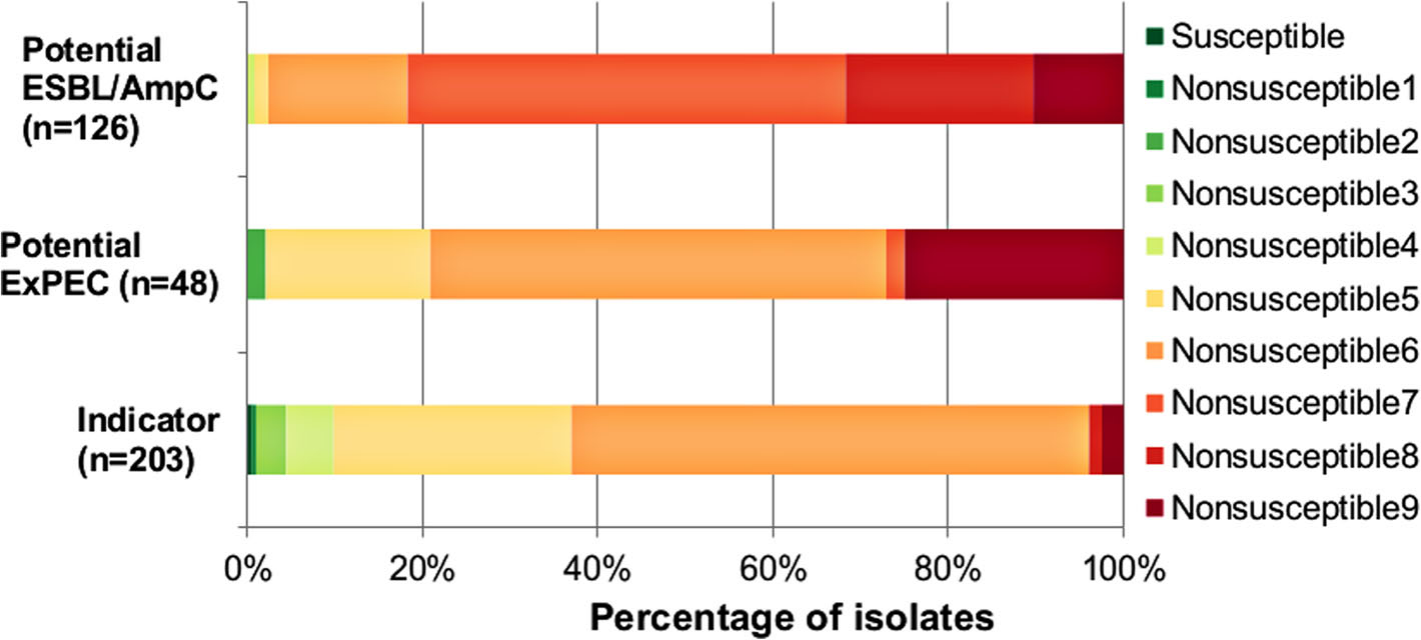
Distribution (%) of indicator (*n* = 203), potential ExPEC (*n* = 48) and potential ESBL/AmpC (*n* = 126) isolates from healthy chickens in Vietnam according to nonsusceptibility profiles. Susceptible: susceptible to all classes of antimicrobials; Nonsusceptible 1 to 9: nonsusceptible to 1 up to 9 classes of antimicrobials; isolates nonsusceptible to 3 up to 7 antimicrobials were considered to be multidrug resistant (MDR), isolates nonsusceptible to 8 or 9 antimicrobials were considered to be possibly extensively drug resistant (XDR)

Almost all indicator isolates [201 (99.0%; 95%CI = 97.2–100)] were multidrug-resistant (MDR, nonsusceptible to three or more antimicrobial classes). Nonsusceptibility to five or six classes of antimicrobials (MDR5 or MDR6) was the most frequent (86.2%); some indicator isolates were even considered as possible XDR (*i.e* extensively drug resistant, isolates that remain susceptible to a maximum of two classes of antimicrobials) (Fig. 1). In the specific collections, isolates were almost all nonsusceptible to more than four classes of antimicrobial, with 31.7 and 25.0% of potential ESBL/AmpC and potential ExPEC isolates being possible XDR, respectively (Fig. 1).

Based on the ECDC’s criteria [22], presumptive ESBL/AmpC-producer isolates were found in 80.0% of the studied farms and 76.2% of potential ESBL/AmpC isolates (Table 2). 3GC-nonsusceptible potential ESBL/AmpC isolates were found in 31 samples whereas 3GC-nonsusceptible indicator *E. coli* isolates were only found in 8 samples.

**Table 2:** Prevalence of presumptive ESBL/AmpC-producers in *Escherichia coli* isolated from healthy chickens in Vietnam following enrichment with ceftriaxone

Unit of study	No. examined	No. of units with positive culture	No. (%; 95%CI) of units carrying presumptive ESBL/AmpC-producer isolates
Isolates	126	N/A	96 (76.2%; 67.8–84.5)
Farms	5	4	4 (80.0%; 28.4–99.4)

*No.* Number, *95%CI* 95% confidence interval, *Presumptive ESBL* isolate resistant to ceftriaxone and/or ceftiofur and susceptible to amoxicillin clavulanic acid and cefoxitine, *Presumptive AmpC* isolate resistant to ceftriaxone in addition to being resistant to amoxicillin clavulanic acid and cefoxitine, *Presumptive ESBL/AmpC-producers* cumulative of presumptive ESBL-producers and presumptive AmpC-producers, *N/A* Not applicable as no culture was done

### Prevalence of antimicrobial resistance genes

All tested indicator isolates (*n* = 80) were carriers of at least one AMR gene with the maximum of 5 AMR genes. The most prevalent AMR genes were *bla*_*TEM*_ (100%; 95%CI = 95.5–100), *tetA* (87.5%; 95%CI = 71.4–100), *aadA1* (71.3%; 95%CI = 56.4–86.1), and *dfrA5* (32.5%; 95%CI = 11.9–53.1) (Table 3). In addition, 36 (61.0%; 95%CI = 34.3–87.7) of potential ESBL/AmpC isolates and 1 (2.1%; 95%CI = 0.0–8.5) potential ExPEC isolate were *bla*_*CTX-M*_-positive; these frequencies for *bla*_*CMY-2*_ were 23 (39.0%; 95%CI = 12.2–65.7) in potential ESBL/AmpC isolates and 4 (8.3%; 95%CI = 0.0–25.6) in potential ExPEC isolates. Sequencing of 30 *bla*_*CTX-M*_-positive isolates showed that this gene was of groups CTX-M-1, − 8 and − 9 and of genotypes *bla*_*CTX-M*-1_ (4 isolates), *bla*_*CTX-M*-14_ (6 isolates), *bla*_*CTX-M*-15_ (5 isolates), *bla*_*CTX-M*-17_ (6 isolates), *bla*_*CTX-M*-57_ (8 isolates) and *bla*_*CTX-M*-87_ (1 isolate). Except for *bla*_*CTX-M*-1_ that was only detected in isolates from farms 4 and 5, the other major genotypes were each found in 3 farms, 1, 4 and 5.

**Table 3:** Prevalence of AMR genes and the 95%CI in 80 indicator isolates

AMR gene	No. (%) of positive isolates	95% CI	Target antimicrobials
*tetA*	70 (87.5)	71.4–100	Tetracycline
*tetB*	7 (8.8)	0.0–21.1	
*dfrA1*	3 (3.8)	0.0–8.1	Trimethoprim-sulfamethoxazole, sulfamethoxazole
*dfrA5*	26 (32.5)	11.9–53.1	
*dfrA7*	7 (8.8)	0.0–20.3	
*aadA1*	57 (71.3)	56.4–86.1	Streptomycin
*bla*_*TEM*_	80 (100)	95.5–100	Ampicillin
*bla*_*OXA-1*_	3 (3.8)	0.0–10.2	
*qnrB*	2 (2.5)	0.0–6.9	Quinolones

*bla*_*CMY-2*_*, bla*_*SHV*,_ *bla*_*CTX-M*_ and *tetC* genes were not detected in any indicator isolate

*AMR* Antimicrobial resistance, *No*. Number, *%* percentage, *95%CI* 95% confidence interval

### Distribution of virulence genes and phylogroups

**Fig. 2:**
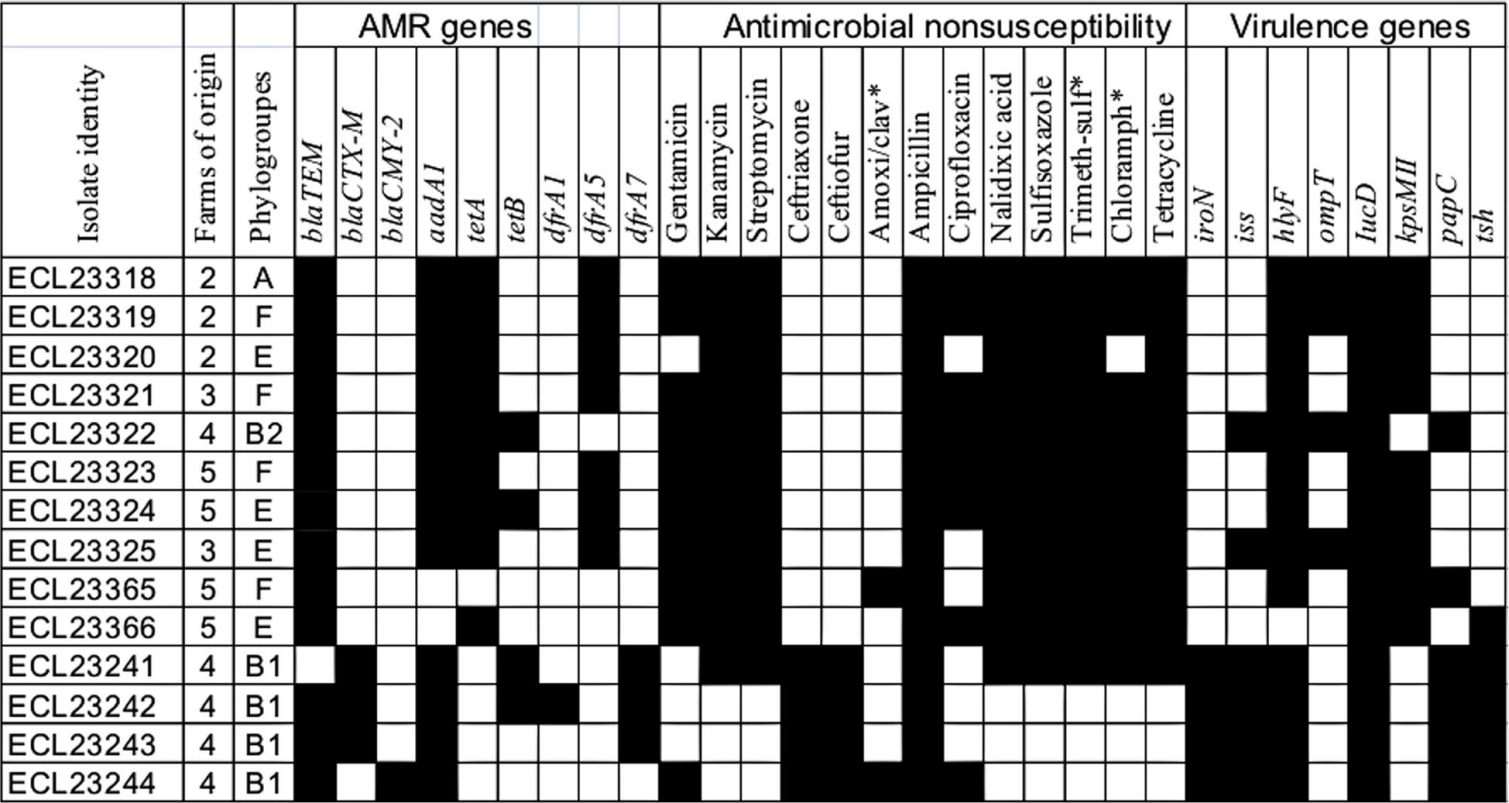
Virulence, phylogenetic groups and AMR profiles of potential ExPEC isolates from healthy chickens in Vietnam. Amoxi/clav: Amoxicillin/clavulanic acid; Trimeth-sulf: Trimethoprim-sulfamethoxazole; Chloramph: Chloramphenicol

The 80 tested indicator isolates were of phylogroups A [32 (40.0%; 95%CI = 20.4–50.5)], B1 [36 (45.0%; 95%CI = 21.4–68.6)], B2 [4 (5.0%; 95%CI = 0.0–14.5)] and D [8 (10.0%; 95%CI = 0.0–26.2)]. Of the 11 tested virulence genes, 8 were detected. In all, 45 (56.3%; 95%CI = 40.2–72.3) indicator *E. coli* isolates were positive to one or more virulence genes; with a maximum of 5 genes. All APEC-associated virulence genes were detected with the prevalence in descending order, *ompT* [23 (28.7%; 95%CI = 11.5–46.0)], *iss* [18 (22.5%; 95%CI = 5.5–39.5)], *hlyF* [15 (18.7%; 95%CI = 1.6–36.2)], *iucD* [14 (17.5%; 95%CI = 0.0–35.8)], *iroN* [10 (12.5%; 95%CI = 4.6–20.4)], and among ExPEC virulence markers, *kpsMII* [5 (6.3%; 95%CI = 0.0–15.4)] and *papC* [2 (2.5%; 95%CI = 0.0–6.4)] were detected. The virulence gene *tsh* [4 (5.0; 95%CI = 0.0–11.9)] was also detected, whereas *cnf*, *sfa/foc* and *afa/dra* were not detected in any isolate. APEC-associated virulence genes were found amongst all of the four phylogroups whereas the *kpsMII* gene associated with ExPEC was mostly detected in isolates of phylogroups B2 and D (data not shown). Twenty-three virulence profiles were found of which the most frequently observed were *ompT* (12 isolates belonging to phylogroup A, B1 and B2), *hlyF* and *iss* (4 isolates each, each mostly of phylogroup B1), *iucD/iss* (3 isolates of phylogroups B1 and D) followed by *iucD/iss/hlyF/ompT*, *iucD* and *iroN/iss/hlyF/ompT* (2 isolates each). Based on genetic criteria proposed by Johnson et al. [23] to define APEC isolates, 44 (55.0%; 95%CI = 38.0–71.9) isolates were positive for at least one of the APEC predictors of which 7 representing 8.8% (95%CI = 2.6–14.9) of the total tested indicator isolates, originating from 4 farms, had four or five of these predictors and thus, were classified as potential virulent APEC. Three (3.7%; 95%CI = 0.0–9.7) indicator isolates, of phylogroups B2 and D, from two farms met criteria defining potential human ExPEC [24]. In the potential ExPEC collection, 17 (35.4%; 95%CI = 0.9–69.9) isolates belonging to phylogroups A, B1 and D were defined as potential virulent APEC and 7 (14.6%; 95%CI = 0.0–31.5), mostly of phylogroup D, were classified as potential human ExPEC. In addition, 4 potential human ExPEC ESBL/AmpC-producers were found in the enriched collection, resulting in a total of 14 isolates potentially capable of infecting humans. Examination of 9 potential human ExPEC belonging to phylogroups B2 (*n* = 1) et D (*n* = 8) using the revisited phylotyping method [25] showed the B2-isolate remains in the same phylogroup, whereas isolates of phylogroup D were reassigned to phylogroup F (*n* = 4) and E (*n* = 4) (Fig. 2). For the other tested isolates (all ESBL/AmpC-producers), for the two B2-isolates, one still belonged to group B2 and the other reassigned to group E, whereas the 9 isolates of phylogroup D were reassigned to phylogroups A (1 isolate), C (1 isolate), E (3 isolates), F (1 isolate) and B2 (3 isolates).

### Prevalence of O serogroups and replicon plasmids, and relationship among ESBL/AmpC-producers

**Fig. 3:**
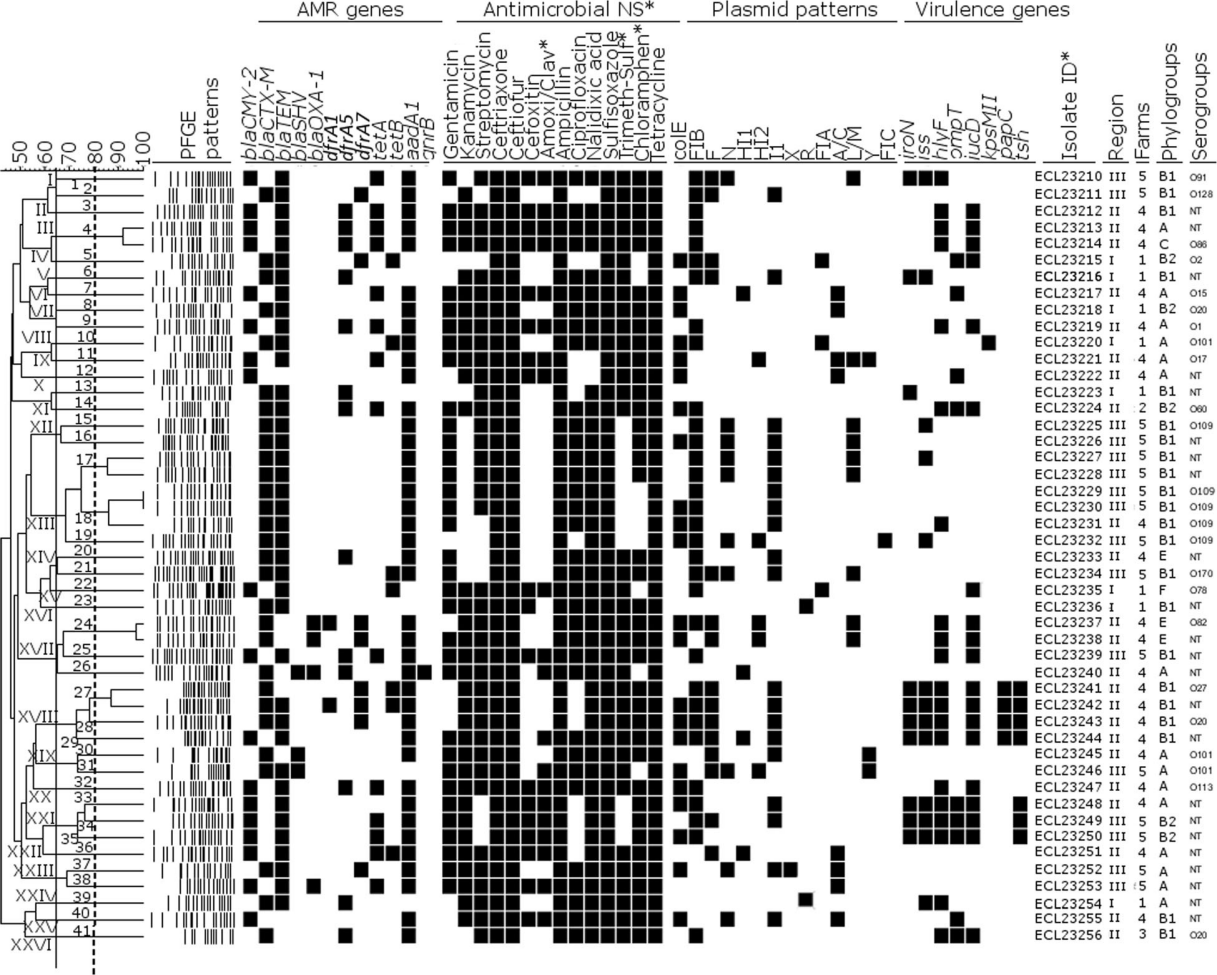
Dendrogram showing the relatedness of 47 *bla*_*CTX-M*_*/bla*_*CMY-2*_-producing *Escherichia coli* isolates from healthy chickens in Vietnam, based on pulsed-field gel electrophoresis (PFGE) patterns. The dendrogram was generated using Dice coefficient and the unweighted pair-group method and arithmetic average (UPGMA). Based on a similarity index of ≥60% (continuous line), 26 majors clusters (I-XXVI) were found inside which 41 PFGE groups (in arabic numerals) were identified when the similarity was set at 80% (discontinued line). AMR: antimicrobial resistance; antimicrobial NS*: antimicrobial nonsusceptibility; Trimeth-sulf: trimethoprim-sulfamethoxazole; Amoxi/clav: Amoxicillin-clavulanic acid; Chloramphen: Chloramphenicol. None of the ESBL/AmpC-producing isolates were positive to AMR gene *tetC* and none was carrier of virulence genes *sfa*, *afa* or *cnf*, then these genes were removed from the dendrogram

Of the 47 ESBL/AmpC-producers tested, only 24 could be assigned to an O-serogroup, with a total of 16 different serogroups observed (Fig. 3). The most frequently observed O serogroups were O109 [5 (10.6%; 95%CI = 0.0–28.8)], followed by O20 [3 (6.4%; 95%CI = 0.0–16.4)] and O101 [3 (6.4%; 95%CI = 2.4–10.4)]. The other serogroups, including O1, O2 and O78, were each observed in one isolate (Fig. 3).

Of the 21 replicon plasmids tested, 14 were detected. All isolates harboured at least one replicon plasmid, presence of replicon plasmids in isolates ranging from 1 to 5. Of these, the most frequently observed were FIB [32 (68.1%; 95%CI = 48.9–87.2)], colE [22 (46.8%; 95%CI = 28.1–65.5)], I1 [20 (42.6%; 95%CI = 15.8–69.2)], and F [15 (31.9%; 95%CI = 22.1–41.7)] (Fig. 3).

PFGE of these 47 ESBL/AmpC-producer isolates revealed their high diversity. Based on similarity level set up 60%, 26 clusters (I - XXVI) were found of which, 11 consisted in at least two isolates (Fig. 3). By setting the similarity between isolates at 80%, 41 PFGE groups (1–41), of which five (PFGE groups 4, 17, 18, 24 and 27) included at least two isolates, were observed within the clusters. Only the PFGE group 18, which includes 3 isolates, the largest number of isolates per group, consisted of isolates from the same phylogroup / serogroup (B1 / O109) and from different farms (Farms 4 and 5) located in two separate regions (Fig. 3). The other major PFGE groups each consisted of two isolates originating from the same farm. In addition, isolates from some clusters shared some similarities in AMR and/or virulence profiles. This was the case for cluster XVIII (which includes the PFGE group 27) of which isolates were positive for AMR genes *bla*_*CTX-M*_, *bla*_*TEM*_, *aadA1*, being nonsusceptible to kanamycin, streptomycin, ceftriaxone, ceftiofur, ampicillin, nalidixic acid, sulfisoxazole, trimethoprim-sulfamethoxazole, chloramphenicol and tetracycline. These XVIII cluster isolates were also positive for replicon plasmids colE, FIB, F and I1 and the virulence genes *iucD*, *papC*, *iroN*, *iss* and *hlyF*, permitting these isolates to be considered both potential virulent APEC and potential human ExPEC. Cluster XXI includes *bla*_*CMY-2*_-, *bla*_*TEM*_- and *aadA1*-positive isolates nonsusceptible to gentamicin, kanamycin, ceftriaxone, ceftiofur, cefoxitin, amoxicillin/clavulanic acid, ampicillin, nalidixic acid, sulfisoxazole, chloramphenicol and tetracycline and positive for the *iroN*, *iss*, *hlyF*, *ompT*, *iucD* and *tsh* virulence genes (Fig. 3) and have been defined as potential virulent APEC.

### Mutations in the quinolone resistance-determining region (QRDR) of *gyrA* and *parC* genes

Two mutation positions were observed in each QRDR gene (Table 4). In *gyrA*, the most prevalent mutation was the substitution of Serine (Ser) by Leucine (Leu) at position 83 [45 (90.0; 95%CI = 84.2–95.7)] and the substitution of Aspartic acid (Asp) by Asparagine (Asn) at position 83 [34 (68.0; 95%CI = 57.5–78.5)]. In *parC* gene, the most frequently observed mutation was the substitution of serine by isoleucine (Ile) at position 80 [42 (84.0; 95%CI = 72.5–95.5)]. Twenty-eight (56.0%; 95%CI = 43.8–68.2) isolates demonstrated a combination of the 3 same mutations, Ser83Leu and Asp87Asn in *gyrA* and Ser80Ile in *parC* and these isolates were from the 5 studied farms. In addition, 41 (82.0%; 95%CI = 69.9–94.1) isolates were carriers of double-serine mutation (*gyrA* Ser83Leu and *parC* Ser80Ile), whereas 38 (76.0%; 95%CI = 65.6–86.41) isolates were carriers of at least two mutations in *gyrA* and one in *parC*.

**Table 4:** Presence of mutations in *gyrA* and *parC* genes of ciprofloxacin-resistant *Escherichia coli* isolated from broilers in Vietnam

Amino acid changes in *gyrA*		Amino acid changes in *parC*		No. of isolates carrying mutation(s) (% and 95CI)	No. of farms of origin
Ser83	Asp87	Ser80	Glu84		
Leu	Asn	Ile	–	28 (56.0; 43.8 – 68.2)	5
Leu	Asn	Ile	Gly	4 (8.0; 0.0–17.3)	3
Leu	–	Ile	–	3 (6.0; 0.0–13.0)	3
Leu	Gly	Ile	–	3 (6.0; 0.0–16.4)	2
Leu	–	–	–	2 (4.0; 0.0–13.9)	1
Leu	Tyr	Ile	–	2 (4.0; 0.0–11.2)	2
Leu	His	Ile	–	1 (2.0; 0.0–6.9)	1
Leu	Arg	–	–	1 (2.0; 0.0–7.4)	1
Leu	Asn	–	–	1 (2.0; 0.0–8.6)	1
–	Asn		–	1 (2.0; 0.0–7.4)	1
–	–	Ile	–	1 (2.0; 0.0–6.9)	1
–	–	–	–	3 (6.0; 0.0–15.5)	3
Total of tested isolates and farms				*50*	*5*

*Ser* serine, *Leu* leucine, *Tyr* tyrosine, *Asp* aspartic acid, *Asn* asparagine, *Ile* isoleucine, *Arg* arginine, *Glu* glutamic acid, *Lys* lysine

### Transferability of ESBL/AmpC genes

The AMR gene transfer experiments were successful for 20 isolates including 3 *bla*_*CMY-2*_ and 17 *bla*_*CTX-M*_ isolates. The *bla*_*CMY-2*_ gene was located on replicon plasmids A/C (2 isolates) and I1 (1 isolate) whereas *bla*_*CTX-M*_ genes were found on replicon plasmids I1 (12 isolates), FIB (3 isolates), and R and HI1 (1 isolate each) (Table 5). A/C plasmids carrying *bla*_*CMY-2*_ co-transferred resistance to gentamicin, chloramphenicol, sulfisoxazole and tetracycline; I1 plasmid-carrying *bla*_*CTX-M*_ co-transferred resistance to tetracycline, sulfisoxazole, trimethoprim-sulfamethoxazole, tetracycline or gentamicin, and the plasmids FIB, R and HI1 carrying *bla*_*CTX-M*_ co-transferred resistance to gentamicin, chloramphenicol, trimethoprim-sulfamethoxazole, sulfisoxazole or tetracycline (Table 5). In addition, in 2 isolates, the *bla*_*CTX-M*_ gene was co-transferred with the *bla*_*TEM*_ gene and in one isolate, *bla*_*CTX-M*_ was co-transferred with *bla*_*SHV*_ (Table 5).

**Table 5:** Characteristics of 20 ESBL/AmpC transformants showing their transferred ESBL/AmpC genes and replicon plasmids, co-transferred AMR and Phylogroup/serogroup, PFGE group and farms origin of the wild-type strains

Transformant ID	Phylogroup/serogroup of the wild-type strains	PFGE group of the wild-type strains	ESBL/AmpC genes transferred	Co-transferred AMR^a^	Plasmid replicon types	Farms origin of the wild-type strains
ECL23217	A/O15	7	*bla*_CMY-2_	CHL, SSS, TET	A/C	4
ECL23356	B1/NT	NA	*bla*_*CTX-M-17,*_ *bla*_*TEM*_	–	I1	5
ECL23216	B1/NT	6	*bla*_*CTX-M-57*_	–	I1	1
ECL23241	B1/O27	27	*bla*_*CTX-M-57*_	–	I1	4
ECL23242	B1/NT	27	*bla*_*CTX-M-57*_	–	I1	4
ECL23243	B1/O20	28	*bla*_*CTX-M-57*_	–	I1	4
ECL23244	B1/NT	29	*bla*_CMY-2_	–	I1	4
ECL23240	A/NT	26	*bla*_*CTX-M-15*_	–	FIB	4
ECL23237	D/O82	24	*bla*_*CTX-M-15*_	–	I1	4
ECL23233	D/NT	20	*bla*_*CTX-M-1*_	SSS, TET	I1	4
ECL23234	B1/O170	21	*bla*_*CTX-M-1*_	SSS, TET	I1	5
ECL23254	A/NT	39	*bla*_*CTX-M-57,*_ *bla*_*TEM*_	TET	R	1
ECL23236	B1/NT	23	*bla*_*CTX-M-57*_	SSS, TET	HI1	1
ECL23229	B1/O109	18	*bla*_*CTX-M-17*_	GEN	I1	5
ECL23220	A/O101	10	*bla*_*CTX-M-17*_	GEN, CHL, SSS, TET	FIB	1
ECL23221	A/O17	11	*bla*_CMY-2_	GEN, CHL, SSS, TET	A/C	4
ECL23223	B1/NT	13	*bla*_*CTX-M-57*_	–	I1	1
ECL23245	A/O101	30	*bla*_*CTX-M-15,*_ *bla*_*SHV*_	–	I1	4
ECL23252	A/NT	37	*bla*_*CTX-M-57*_	SXT, SSS	I1	5
ECL23256	B1/O20	41	*bla*_*CTX-M-1*_	TET	FIB	5

^a^*bla*_*CTX-M*_ transformants were nonsusceptible to ceftriaxone, ceftiofur and ampicillin, and *bla*_*CMY-2*_ transformants were nonsusceptible to ceftriaxone, ceftiofur, amoxicillin/clavulanic acid and ampicillin

## Discussion

This study showed a very high proportion of MDR (99%) in indicator isolates. A likely explanation to this high level of nonsusceptibility could be the extensive use of antimicrobial in chickens in Vietnam, often without veterinary prescription, as reported in many studies [17–19]. Other studies have reported similar levels of AMR in *E. coli* from poultry in Vietnam [26, 27]. In contrast, the level of nonsusceptibility to 3GC was very low (3.9%) in indicator isolates, agreeing with the prevalence of 3.1% of resistance reported in 3GC in Vietnam [27]. However, following enrichment with ceftriaxone, a high prevalence (76.2%) of presumed ESBL/AmpC isolates was observed. It should be noted that in both indicator and potential ESBL/AmpC isolates, 3GC-nonsusceptible isolates were found in 4 out of the 5 studied farms. However, these isolates were recovered from eight samples in the indicator *E. coli* collection whereas they were detected in 31 samples in the enriched collection, clearly demonstrating higher sensitivity of detection of the protocol used in the later case. The significant increase in nonsusceptibility to 3GC, observed in the potential ESBL/AmpC isolates, is indicative of the extent of selection of resistance that may be induced by the use of antimicrobials. However, this prevalence of 76.2% is slightly lower compared to the prevalence of 89.7% of potential ESBL/AmpC isolates resistant to cefotaxime that has been previously reported in Vietnam [26]. This nonsusceptibility to 3GC could also be due to the use of antimicrobials other than 3GC. For instance, the selection of ESBL-producer *E. coli* following aminopenicillin use has been reported [28].

Of the ESBL/AmpC genes examined in indicator isolates, *bla*_*TEM*_ (100%) was the most frequently observed gene whereas neither *bla*_*CTX-M-*_ nor *bla*_*CMY-2*_-positive isolates were detected (Table 3). Likewise, *bla*_*TEM*_ (83.6%) was also the predominant ESBL gene in the potential ESBL/AmpC isolates, followed by *bla*_*CTX-M*_ and *bla*_*CMY-2*_ which were detected in 61.0 and 39.0% of these isolates, respectively. In Brazil, 72% of potential ESBL/AmpC recovered from fecal chicken samples in two farms were found to be *bla*_*CMY-2*_-producers [29]. However, these authors only tested for the presence of AmpC genes among *E. coli* presumed ESBL/AmpC-producers. Our results show a predominance of the CTX-M gene among 3GC-resistant isolates, consistent with the results we obtained for *E. coli* isolates from carcasses in Vietnam [30]. In contrast, in another study on chicken fecal *E. coli* from Quebec (Canada), we found that 3GC-resistance was mainly due to *bla*_*CMY-2*_ rather than *bla*_*CTX-M*_ (unpublished results), and a study in Sweden [31] also found that *bla*_*CMY-2*_ predominated among fecal *E. coli* isolated from broilers. These differences between countries could be related to differences in the types of antimicrobials used in poultry or to differences in the geographical distribution of resistance genes and their variants [32]. In our study, *bla*_*CTX-M*_ genes were of CTX-M-1 and -9 groups and of genotypes *bla*_*CTX-M-1*_, *bla*_*CTX-M*-15_, *bla*_*CTX-M-14*_, *bla*_*CTX-M-17*_, *bla*_*CTX-M-57*_, and *bla*_*CTX-M-87*_. Chicken fecal *E. coli* isolates harbouring *bla*_*CTX-M-1*_ and _− 9_ groups have already been reported in Vietnam [26]. Likewise, *bla*_*CTX-M*_ of genotypes − 1, − 15, − 14, − 17 have been detected in *E. coli* isolated from chickens in Vietnam and China [33, 34]. In addition, *bla*_*CTX-M-55*_, which is identical to *bla*_*CTX-M-57*_ [35], was reported in *E. coli* isolated from Vietnamese chicken farms [34]. The genotype *bla*_*CTX-M-87*_ was first described in an *E. coli* strain isolated from inpatient in China in 2009 [36] and in the best of our knowledge, this is the first report of this variant in *E. coli* isolated from chickens. In our screening of potential ESBL/AmpC isolates of Vietnam for carriage of *mcr-1* or *mcr-2* genes mediating colistin-resistance, we did not detect *mcr-2*, whereas some isolates carried *mcr-1* in association with *bla*_*CTX-M*_ or *bla*_*CMY-2*_ genes (unpublished data). *bla*_*CTX-M-14*_, _− 15_ and _− 55_ have also been identified in Vietnam, sometimes in association with *mcr-1* gene, from chicken farm workers or community individuals [34] or in postsurgical infections from patients [37]. Although the former study [34] did not find any relationship between the *bla*_*CTX-M*_ producing isolates from chickens and humans, our findings demonstrate that further investigation into the possible links between human and poultry isolates is warranted.

Very high prevalence of nonsusceptibility against ciprofloxacin (59.6%) was observed in indicator isolates, all farms harbouring ciprofloxacin resistant isolates. Nguyen et al. [27] also reported high prevalence of resistance to ciprofloxacin in farms (91.8%). These authors also reported that ciprofloxacin resistance was significantly associated with the use of commercial feeds containing antimicrobials, non-compliance with biosecurity measures or the use of quinolones. Sequencing of ciprofloxacin-resistant isolates demonstrated two mutation positions in both *gyrA* and *parC* with all amino acid changes described elsewhere [38–40]. The presence of isolates carrying two mutations in *gyrA* gene and one in *parC* has been suggested as indicative of the high level of resistance demonstrating the widespread use of quinolones [41]. In the other hand, 82.0% of ciprofloxacin-resistant isolates originating from the five farms carried the double-serine mutation in *gyrA* (Ser83Leu) and *parC* (Ser80Ile). This double mutation has been described as a fitness factor that has helped the ST131 pandemic clone to successfully spread into new ecological niches [42], and therefore, these isolates could have a clonal relationship.

It should be noted, however, that the small size of our sample (*n* = 5) and the sampling method used (convenience), the prevalence we report here cannot be inferred to all farms in Vietnam. Nevertheless, prevalence estimates are probably representative of *E. coli* present in these 5 farms at the time of the study. This study is therefore preliminary to a large-scale study that could include more farms from different regions of Vietnam and could be conducted using more sophisticated tools such as whole genome sequencing.

The indicator *E. coli* isolates, as well as isolates producing ESBL/AmpC, in this study were found in the four phylogenetic groups A, B1, B2 and D, although the ESBL-producer isolates were mostly of phylogroup B1. Le et al. [43] also found that ESBL-producer *E. coli* isolated from poultry in Vietnam were mostly of the phylogroup B1. In addition, some MDR isolates of phylogroups B2 and F were carriers of at least two virulence genes associated with human ExPEC, suggesting their potential to cause infections in humans, *E. coli* of phylogroups B2 and F being associated with ExPEC causing infections in humans [25, 44]. In our previous study on clinical chicken *E. coli* isolates from Senegal [40] and in our study on chicken carcasses collected on Vietnamese markets [30], potential human ExPEC isolates expressing nonsusceptibility to several antimicrobials and belonging to phylogroup F were also observed.

Our PFGE analysis revealed a high genetic diversity among ESBL/AmpC-producing isolates, as already shown by other studies [45, 46]. This could suggest that dissemination of ESBL/AmpC genes occurs via plasmids rather than by clones. However, some closely related isolates were observed, as for the PFGE group 18 in cluster XIII, which included three *bla*_*CTX-M*_-producing isolates belonging to phylogroup B1 and serogroup O109 and originating from two separate farms located in two regions. This could suggest a single source of contamination or spread between farms. A common source of contamination could be hatcheries that supply farms with day-old chicks, whereas spread between farms could be linked to movement of workers between farms. In fact, the proximity of poultry farms to human settlements in Vietnam has already constituted a major threat to the transmission of zoonotic diseases [47].

The AMR transfer experiments clearly demonstrated the potential role of plasmids in the spreading of AMR within and between farms. The *bla*_*CMY-2*_ gene was carried by the A/C and I1 plasmids whereas the *bla*_*CTX-M*_ genes were located on the I1, FIB, R and HI1. This is the first study identifying plasmids carrying ESBL/AmpC genes in *E. coli* isolated from fecal chicken samples in Vietnam. Our studies of *E. coli* isolates from fecal samples in healthy chickens from Senegal and Quebec (unpublished data) also demonstrated the location of both *bla*_*CMY-2*_ and *bla*_*CTX-M*_ on I1, although A/C, R and HI1 plasmids carrying these genes were not observed in our studies in Senegal or Quebec, which could be partly due to a different geographical distribution of some plasmids. Some plasmids carrying the ESBL/AmpC genes co-transferred resistance to other antimicrobials and it is possible that this co-localization may have contributed to the high prevalence of AMR through co-selection following the use of these antimicrobials. Moreover, certain plasmids carrying the same beta-lactamase gene were identified in isolates genetically very heterogeneous and originating from different farms, suggesting the spread of these plasmids between farms. I1, carrying either *bla*_*CTX-M*_ or *bla*_*CMY-2*_, was most frequently observed in unrelated isolates, supporting this hypothesis as it is one of the plasmids capable of successfully spreading on a large-scale [48, 49].

## Conclusions

In conclusion, *E. coli* isolated from healthy chicken farms in Vietnam were highly MDR and expressed resistance against critically important antimicrobials in humans such as 3GC and ciprofloxacin. Ciprofloxacin resistance was mainly due to mutations in the *gyrA* and *parC* genes whereas 3GC-resistance was mediated by replicon plasmids bearing *bla*_*CTX-M*_*/bla*_*CMY-2*_. The results demonstrated the potential role of plasmids in the spreading of AMR within and between farms. In addition, some ESBL/AmpC-producing isolates possessed virulence gene profiles which could allow them to cause infections in humans. These results demonstrated the necessity to monitor AMR and control antimicrobial use in poultry in Vietnam.

## Methods

### Sample collection and processing

Faecal samples were collected from five healthy chicken farms chosen by convenience and located in the provinces of Hoa Binh, Thai Nguyen and Bac Giang, in the North of Vietnam. Faecal swabs were randomly collected from five points on each pen floor using sterile cotton swabs (Nam Khoa Biotek Company, Ho Chi Minh city, Vietnam) and pooled together by pen. Depending on the number of pens on farms, 4 to 15 pooled samples per farm were obtained, for a total of 51 pooled feces samples.

All samples were transported to the laboratory at the National Veterinary Institute in Hanoi, Vietnam. After enriching in peptone water at 37 °C, overnight, samples were cultured on MacConkey agar, and incubated at 37 °C, overnight. All samples were kept at 4 °C until shipping to the OIE reference laboratory for *E. coli* (EcL) in Canada.

### Establishment of *E. coli* collections

Collections of indicator (*i.e* isolates selected on MacConkey without enrichment), potential ExPEC (isolates selected on the basis of the possession of at least one of the virulence genes *iucD*, *tsh*, *papC* and *cnf* [13]) and potential ESBL/AmpC isolates (isolates selected on MacConkey agar supplemented with ceftriaxone 1 mg/L [50]) were established (Additional file 1: Figure S1). All isolates selected were confirmed as *E. coli* by the detection, using PCR, of the *uidA* housekeeping gene. PCR conditions used to detect *uidA* gene included initial denaturation (95 °C, 2 mn), 24 cycles of denaturation (94 °C, 30 s), annealing (65 °C, 30 s), extension (72 °C, 30 s), and final extension (4 °C).

### Antimicrobial susceptibility testing

Isolates of the three collections were examined for their susceptibility against 14 antimicrobials of nine classes using the disk diffusion method (Kirby-Bauer) [51]. Breakpoints were those recommended by the Clinical and Laboratory Standards Institute (CLSI) in 2016 [52] for most of the antimicrobials and in 2015 for ceftiofur [53]**.** The *E. coli* strain ATCC 25922 was used as quality control strain.

### Antimicrobial resistance genes

Eighty (80) isolates randomly selected from the indicator collection and originating from the 51 samples and all potential ExPEC isolates were examined by PCR for 13 AMR genes including streptomycin (*aadA1*), tetracycline (*tetA*, *tetB* and *tetC*), trimethoprim-sulfamethoxazole (*dfrA1*, *dfrA5* and *dfrA7*), fluoroquinolones (*qnrB*) and β-lactams (*bla*_*TEM*_, *bla*_*SHV*_, *bla*_*OXA-1*_, *bla*_*CTX-M*_ and *bla*_*CMY-2*_). In addition, 59 potential ESBL/AmpC randomly selected per sample among 108 3GC-nonsusceptible isolates were tested for the presence of beta-lactamase genes (*bla*_*TEM*_, *bla*_*SHV*_, *bla*_*OXA-1*_, *bla*_*CTX-M*_ and *bla*_*CMY-2*_). All of these tests were performed as described in our previous study [40]. In addition, 30 *bla*_*CTX-M*_-positive isolates, originated from the five farms, were randomly selected and tested by PCR for CTX-M-1, − 2, − 8 and − 9 groups [54, 55]. DNA of these isolates was purified, sequenced and the sequence analysis was performed as described in our previous study [40].

### Detection of mutations in the quinolone-resistance determining region (QRDR)

The regions of *gyrA* and *parC* genes in QRDR were amplified by PCR as described previously [56], in 50 ciprofloxacin-resistant (one randomly selected isolate in each of the 50 samples harboring ciprofloxacin resistant isolates). DNA purification, sequencing and sequence analysis were performed as described in our previous study [40].

### Virulence genes and phylogenetic groups

All isolates tested above for AMR genes also were examined by PCR for 11 virulence genes including those associated with APEC [23] or human ExPEC isolates [57]. Each isolate was also examined by PCR to be assigned to one of the four main phylogenetic groups A, B1, B2 and D [58]. In addition, isolates belonging to phylogroups B2 or D, producers of ESBL/AmpC and/or classified potential human ExPEC, were tested by the revised phylotyping method [25]. Primers used for the PCRs performed in this study and the thermal conditions are available in our previous study [40].

### Serotyping

Fourty-seven randomly selected *bla*_*CTX-M*_- or *bla*_*CMY-2*_-positive isolates, originating from the five farms, were tested by standard agglutination methods [59] to detect 86 O-serogroups described on the EcL website [60].

### Pulsed field gel electrophoresis (PFGE)

In order to estimate their clonal relationship, the 47 isolates previously screened for serogroups were sub-typed by PFGE using *Xba*I-restriction enzyme [61]. The similarities of fragments were compared using a Dice coefficient at 1% tolerance and 0.5% optimization, and a dendrogram was generated in BioNumerics (Applied Maths) software (v. 6.6) using the unweighted pair group method with arithmetic mean (UPGMA) clustering method. Clusters were defined as isolates sharing at least 60% of similarity (cut-off value) [62] as estimated by BioNumerics from the dendogram and PFGE groups as isolates sharing at least 80% of similarity [63].

### Replicon typing and AMR transferability

The presence of plasmids of the different incompatibility groups was examined in the 47 isolates using PCR based replicon typing as described [64]. Purified plasmid DNA of 30 randomly selected ESBL/AmpC-producer isolates was electroporated into *E. coli* DH10B Electromax™ competent cells (Invitrogen, Calsbad, CA).

Transformants were selected on Mueller Hinton agar supplemented with ceftriaxone 2 μg/ml [65]. Up to five transformants, when available, were screened by PCR for the presence of incompatibility plasmid and for all AMR genes present in the corresponding wild type strains. Transformants carrying ESBL/AmpC genes were subsequently tested for their susceptibility to the 14 antimicrobials as mentioned above.

### Statistical analysis

Prevalence of AMR was estimated at the isolate and farm levels. A farm was considered as resistant for an antimicrobial when at least one resistant isolate was detected for this antimicrobial. In addition, prevalence of virulence and AMR genes, phylogenetic groups, serogroups and plasmids were estimated in indicator and potential ExPEC isolates, whereas the prevalence of *bla*_*CMY-2*_- and *bla*_*CTX-M*_-positive isolates was estimated in selected potential ESBL/AmpC isolates. All prevalence estimates and 95% confidence limits at the isolate level were adjusted for potential clustering within farms and, when a subset of isolates was selected, for sampling weights. At the farm level, exact confidence limits were estimated. Statistical analyses were performed in SAS version 9.4 using the Freq or Surveyfreq procedure.

## Additional file

Additional file 1: **Figure S1.** Methodological approach used in this study. (PDF 208 kb)

## Abbreviations

3GC: Third-generation cephalosporins
AmpC: Cephamycinase
AMR: Antimicrobial resistance
APEC: Avian pathogenic *E. coli*
CI: Confidence interval
EcL: OIE reference laboratory for *E. coli*
ESBL: Extended-spectrum beta-lactamases
ExPEC: Extraintestinal pathogenic *E. coli*
MDR: Multidrug resistance
OIE: World Animal Health Organisation
PFGE: Pulsed Field Gel Electrophoresis
QRDR: Quinolone-resistance determining region
UPGMA: Unweighted pair group method with arithmetic mean
WHO: World Health Organization.
XDR: Extensively drug resistance

## References

[1] Erb A, Stürmer T, Marre R, Brenner H (2007). Prevalence of antibiotic resistance in *Escherichia coli:* overview of geographical, temporal, and methodological variations. Eur J Clin Microbiol Infect Dis.

[2] WHO [Whorld Health Organization]. Antimicrobial Resistance: Global report on Surveillance. https://apps.who.int/iris/bitstream/handle/10665/112642/9789241564748_eng.pdf;jsessionid=B63CEBE5E198C4C21BEEF83739505411?sequence=1. Accessed 5 June 2018.

[3] Geser N, Stephan R, Hächler H (2012). Occurrence and characteristics of extended-spectrum β-lactamase (ESBL) producing *Enterobacteriaceae* in food producing animals, minced meat and raw milk. BMC Vet Res.

[4] Woodford N, Ward ME, Kaufmann ME, Turton J, Fagan EJ, James D, Johnson AP, Pike R, Warner M, Cheasty T, Pearson A, Harry S, Leach JB, Loughrey A, Lowes JA, Warren RE, Livermore DM (2004). Community and hospital spread of *Escherichia coli* producing CTX-M extended-spectrum β-lactamases in the UK. J Antimicrob Chemother.

[5] Brinas L, Moreno MA, Zarazaga M, Porrero C, Sáenz Y, García M, Dominguez L, Torres C (2003). Detection of CMY-2, CTX-M-14, and SHV-12 β-lactamases in *Escherichia coli* fecal-sample isolates from healthy chickens. Antimicrob Agents Chemother.

[6] Randall L, Clouting C, Horton RA, Coldham NG, Wu G, Clifton-Hadley FA, Davies RH, Teale CJ (2010). Prevalence of *Escherichia coli* carrying extended-spectrum β-lactamases (CTX-M and TEM-52) from broiler chickens and turkeys in Great Britain between 2006 and 2009. J Antimicrob Chemother.

[7] Kuo H-C, Chou C-C, Tu C, Gong S-R, Han C-L, Liao J-W, Chang S-K (2009). Characterization of plasmid-mediated quinolone resistance by the *qnrS* gene in *Escherichia coli* isolated from healthy chickens and pigs. Vet Med.

[8] Laube H, Friese A, von Salviati C, Guerra B, Käsbohrer A, Kreienbrock L, Roesler U (2013). Longitudinal monitoring of extended-spectrum-beta-lactamase/AmpC-producing *Escherichia coli* at German broiler chicken fattening farms. Appl Environ Microbiol.

[9] Costa D, Vinue L, Poeta P, Coelho AC, Matos M, Saenz Y, Somalo S, Zarazaga M, Rodrigues J, Torres C (2009). Prevalence of extended-spectrum beta-lactamase-producing *Escherichia coli* isolates in faecal samples of broilers. Vet Microbiol.

[10] Bevan ER, Jones AM, Hawkey PM (2017). Global epidemiology of CTX-M β-lactamases: temporal and geographical shifts in genotype. J Antimicrob Chemother.

[11] Bortolaia V, Larsen J, Damborg P, Guardabassi L (2011). Potential pathogenicity and host range of extended-spectrum β-lactamase-producing *Escherichia coli* isolates from healthy poultry. Appl Environ Microbiol.

[12] Stacy AK, Mitchell NM, Maddux JT, De la Cruz MA, Duran L, Giron JA, Curtuss R, Mellata M (2014). Evaluation of the prevalence and production of *Escherichia coli* common pilus among avian pathogenic *E. coli* and its role in virulence. PLoS One.

[13] Stromberg ZR, Johnson JR, Fairbrother JM, Kilbourne J, Van Goor A, Curtiss R 3rd, Mellata M. Evaluation of *Escherichia coli* isolates from healthy chickens to determine their potential risk to poultry and human health. PLoS One. 2017;12:e0180599 https://journals.plos.org/plosone/article?id=10.1371/journal.pone.0180599. 10.1371/journal.pone.0180599 PMC5495491 28671990

[14] Bergeron CR, Prussing C, Boerlin P, Daignault D, Dutil L, Reid-Smith J, Zhanel GG, Manges AR (2012). Chicken as reservoir for extraintestinal pathogenic *Escherichia coli* in humans, Canada. Emerg Infect Dis.

[15] Carrique- Mas JJ, Trung NV, Hoa NT, Mai HH, Thanh TH, Campbell JI, Wagenaar JA, Hardon A, Hieu TQ, Schultsz C (2015). Antimicrobial usage in chicken production in the Mekong Delta of Vietnam. Zoonoses Pub Health.

[16] Van Cuong N, Nhung NT, Nghia NH, Mai Hoa NT, Trung NV, Thwaites G, Carrique- Mas J (2016). Antimicrobial consumption in medicated feeds in vietnamese pig and poultry production. EcoHealth..

[17] Nhung NT, Cuong NV, Thwaites G, Carrique- Mas J (2016). Antimicrobial usage and antimicrobial resistance in animal production in Southeast Asia: a review. Antibiotics..

[18] Usui M, Ozawa S, Onozato H, Kuge R, Obata Y, Uemae T, Ngoc PT, Heriyanto A, Chalemchaikit T, Makita K, Muramatsu Y, Tamura Y (2014). Antimicrobial susceptibility of indicator bacteria isolated from chickens in southeast Asian countries (Vietnam, Indonesia and Thailand). Vet Med Sci.

[19] Nhung N, Cuong NV, Campbell J, Hoa NT, Bryant JE, Truc VN, Kiet BT, Trung NV, Hien VB, Thwaites G, Baker S, Carrique-Mas J (2015). High levels of antimicrobial resistance among *Escherichia coli* isolates from livestock farms and synanthropic rats and shrews in the Mekong Delta of Vietnam. Appl Environ Microbiol.

[20] Magiorakos AP, Srinivasan A, Carey RB, Carmeli Y, Falagas ME, Giske CG, Harbarth S, Hindler JF, Kahlmeter G, Olsson-Liljequist B, Paterson DL, Rice LB, Stelling J, Struelens MJ, Vatopoulos A, Weber JT, Monnet DL (2012). Multidrug-resistant, extensively drug-resistant and pandrug-resistant bacteria: an international expert proposal for interim standard definitions for acquired resistance. Clin Microbiol Infect.

[21] Sweeney MT, Lubbers BV, Watts JL (2018). Applying definitions for multidrug resistance, extensive drug resistance and pandrug resistance to clinically significant livestock and companion animal bacterial pathogens. J Antimicrob Chemother.

[22] EFSA [European Food Safety Authority] and ECDC [European Centre for Disease Prevention and Control] (2018). The European Union summary report on antimicrobial resistance in zoonotic and indicator bacteria from humans, animals and food in 2016. EFSA J.

[23] Johnson TJ, Wannemuehler Y, Doetkott C, Johnson SJ, Rosenberger SC, Nolan LK (2008). Identification of minimal predictors of avian pathogenic *Escherichia coli* virulence for use as a rapid diagnostic tool. J Clin Microbiol.

[24] Johnson JR, Murray AC, Gajewski A, Sullivan M, Snippes P, Kuskowski MA, Smith KE (2003). Isolation and molecular characterization of nalidixic acid-resistant extraintestinal pathogenic *Escherichia coli* from retail chicken products. Antimicrob Agents Chemother.

[25] Clermont O, Christensen JK, Denamur E, Gordon DM (2013). The Clermont *Escherichia coli* phylo-typing method revisited: improvement of specificity and detection of new phylo-groups. Environ Microbiol Rep.

[26] Nguyen DP, Nguyen TA, Le TH, Tran NM, Ngo TP, Dang VC, Kawai T, Kamki T, Kawahara R, Jinnai M, Yonogi S, Hirai Y, Yamamoto Y, Kumeda Y (2016). Dissemination of extended-spectrum β-lactamase-and AmpC β-lactamase-producing *Escherichia coli* within the food distribution system of Ho Chi Minh City, Vietnam. Biomed Res Int.

[27] Nguyen VT, Carrique-Mas JJ, Ngo TH, Ho HM, Ha TT, Campbell JI, Nguyen TN, Hoang NN, Pham VM, Wagenaar JA, Hardon A, Thai QH, Schultsz C (2015). Prevalence and risk factors for carriage of antimicrobial-resistant *Escherichia coli* on household and small-scale chicken farms in the Mekong Delta of Vietnam. J Antimicrob Chemother.

[28] Agersø Y, Jensen JD, Hasman H, Pedersen K (2014). Spread of extended spectrum cephalosporinase-producing *Escherichia coli* clones and plasmids from parent animals to broilers and to broiler meat in a production without use of cephalosporins. Foodborne Pathog Dis.

[29] Ferreira JC, Penha Filho RAC, Andrade LN, Berchieri Junior A, Darini ALC (2017). Diversity of plasmids harboring blaCMY-2 in multidrug-resistant *Escherichia coli* isolated from poultry in Brazil. Diagn Microbiol Infect Dis.

[30] Sary K, Fairbrother JM, Arsenault J, de LAgarde M, Boulianne M. Antimicrobial resistance and virulence gene profiles among *Escherichia coli* isolates from retail chicken carcasses in Vietnam. Foodborne Pathog Dis. 2019. 10.1089/fpd.2018.2555. 10.1089/fpd.2018.2555 30767657

[31] Bengtsson B (2009). Swedish veterinary antimicrobial resistance monitoring (SVARM) 2008*.* Svensk Veterinärtidning.

[32] Baron S, Jouy E, Larvor E, Eono F, Bougeard S, Kempf I (2014). Impact of third-generation cephalosporin administration in hatcheries on fecal *E. coli* antimicrobial resistance in broilers and layers. Antimicrob Agents Chemother.

[33] Li Y, Chen L, Wu X, Huo S (2015). Molecular characterization of multidrug-resistant avian pathogenic *Escherichia coli* isolated from septicemic broilers. Poult Sci.

[34] Bui TKN, Bui TMH, Ueda S, Le DT, Yamamoto Y, Hirai I (2017). Potential transmission opportunity of CTX-M-producing *Escherichia coli* in large-scale chicken farm in Vietnam. J Glob Antimicrob Resist.

[35] Cortés P, Blanc V, Mora A, Dahbi G, Blanco JE, Blanco M, Lopez C, Andreu A, Navarro F, Alonso MP, Bou G, Blanco J, Llagostera M (2010). Isolation and characterization of potentially pathogenic antimicrobial-resistant *Escherichia coli* strains from chicken and pig farms in Spain. Appl Environ Microbiol.

[36] Yin J, Cheng J, Sun Z, Ye Y, Gao YF, Li JB, Zhang XJ (2009). Characterization of two plasmid-encoded cefotaximases found in clinical *Escherichia coli* isolates*:* CTX-M-65 and a novel enzyme, CTX-M-87. J Med Microbiol.

[37] Biedenbach DJ, Bouchillon SK, Hoban DJ, Hackel M, Phuong DM, Nga TT, Phuong NT, Phuong TT, Badal RE (2014). Antimicrobial susceptibility and extended-spectrum beta-lactamase rates in aerobic gram-negative bacteria causing intra-abdominal infections in Vietnam: report from the study for monitoring antimicrobial resistance trends (SMART 2009–2011). Diagn Microbiol Infect Dis.

[38] Fu Y, Zhang W, Wang H, Zhao S, Chen Y, Meng F, Zhang Y, Xu H, Chen X, Zhang F (2013). Specific patterns of *gyrA* mutations determine the resistance difference to ciprofloxacin and levofloxacin in *Klebsiella pneumoniae* and *Escherichia coli*. BMC Infect Dis.

[39] McDonald LC, Chen FJ, Lo HJ, Yin HC, Lu PL, Huang CH, Chen P, Lauderdale TL, Ho M (2001). Emergence of reduced susceptibility and resistance to fluoroquinolones in *Escherichia coli* in Taiwan and contributions of distinct selective pressures. Antimicrob Agents Chemother.

[40] Vounba P, Kane Y, Ndiaye C, Arsenault J, Fairbrother JM, Bada Alambédji R (2018). Molecular characterization of *Escherichia coli* isolated from chickens with Colibacillosis in Senegal. Foodborne Pathog Dis.

[41] Vanni M, Meucci V, Tognetti R, Cagnardi P, Montesissa C, Piccirillo A, Rossi AM, Di Bello D, Intorre L (2014). Fluoroquinolone resistance and molecular characterization of *gyrA* and *parC* quinolone resistance-determining regions in *Escherichia coli* isolated from poultry. Poult Sci.

[42] Fuzi M, Szabo D, Csercsik R (2017). Double-serine fluoroquinolone resistance mutations advance major international clones and lineages of various multi-drug resistant Bacteria. Front Microbiol.

[43] Le QP, Ueda S, Nguyen TN, Dao TV, Van Hoang TA, Tran TT, Hirai I, Nakayama T, Kawahara R, Do TH, Vien QM, Yamamoto Y (2015). Characteristics of extended-spectrum β-lactamase–producing *Escherichia coli* in retail meats and shrimp at a local market in Vietnam. Foodborne Pathog Dis.

[44] Vangchhia B, Abraham S, Bell JM, Collingnon P, Gison JS, Ingram PR, Johnson JR, Kennedy K, Trott DJ, Turnidge JD, Gordon DM (2016). Phylogenetic diversity, antimicrobial susceptibility and virulence characteristics of phylogroup F *Escherichia coli* in Australia. Microbiol..

[45] Randall LP, Clouting C, Horton RA, Coldham NG, Wu G, Clifton-Hadley FA, Davies RH, Teale CJ (2010). Prevalence of *Escherichia coli* carrying extended-spectrum β-lactamases (CTX-M and TEM-52) from broiler chickens and turkeys in Great Britain between 2006 and 2009. J Antimicrob Chemother.

[46] Girlich D, Poirel L, Carattoli A, Kempf I, Lartigue MF, Bertini A, Nordmann P (2007). Extended-spectrum β-lactamase CTX-M-1 in *Escherichia coli* isolates from healthy poultry in France. Appl Environ Microbiol.

[47] Burgos S, Hong Hanh PT, Roland-Holst D, Burgos SA (2007). Characterization of poultry production systems in Vietnam. Int J Poult Sci.

[48] Carattoli A (2011). Plasmids in gram negatives: molecular typing of resistance plasmids. Int J Med Microbiol.

[49] Carattoli A (2009). Resistance plasmid families in *Enterobacteriaceae*. Antimicrob Agents Chemother.

[50] Agersø Y, Aarestrup FM, Pedersen K, Seyfarth AM, Struve T, Hasman H (2012). Prevalence of extended-spectrum cephalosporinase (ESC)-producing *Escherichia coli* in Danish slaughter pigs and retail meat identified by selective enrichment and association with cephalosporin usage. J Antimicrob Chemother.

[51] CLSI (2018). [clinical and laboratory standards institute]. Performance standards for antimicrobial disk and dilution susceptibility tests for bacteria isolated from animals. 5th Ed. CLSI standard VET01.

[52] CLSI [Clinical and Laboratory Standards Institute] (2016). Performance Standards for Antimicrobial Susceptibility Testing. 26th Ed. CLSI supplement M100S.

[53] CLSI [Clinical and Laboratory Standards Institute] (2015). Performance Standards for Antimicrobial Disk and Dilution Susceptibility Tests for Bacteria Isolated from Animals. CLSI supplement VET01S.

[54] Borgogna TR, Borgogna JL, Mielke JA, Brown CJ, Top EM, Botts RT, Cummings DE (2016). High diversity of CTX-M extended-spectrum β-lactamases in municipal wastewater and urban wetlands. Microb Drug Resist.

[55] Paauw A, Fluit AC, Verhoef J, Leverstein-van Hall MA (2006). *Enterobacter cloacae* outbreak and emergence of quinolone resistance gene in Dutch hospital. Emerg Infect Dis.

[56] Everett MJ, Jin YF, Ricci V, Piddock LJ (1996). Contributions of individual mechanisms to fluoroquinolone resistance in 36 *Escherichia coli* strains isolated from humans and animals. Antimicrob Agents Chemother.

[57] Johnson JR, Kuskowski MA, Owens K, Gajewski A, Winokur PL (2003). Phylogenetic origin and virulence genotype in relation to resistance to fluoroquinolones and/or extended-spectrum cephalosporins and cephamycins among *Escherichia coli* isolates from animals and humans. J Infect Dis.

[58] Clermont O, Bonacorsi S, Bingen E (2000). Rapid and simple determination of the *Escherichia coli* phylogenetic group. Appl Environ Microbiol.

[59] Orskov I, Orskov F, Jann B, Jann K (1977). Serology, chemistry, and genetics of O and K antigens of *Escherichia coli*. Bacteriol Rev.

[60] EcL [OIE reference laboratory for *E. coli*]. *Escherichia coli* serotyping*.* http://www.ecl-lab.com/en/products/serotyping.asp. Accessed 05 June 2018.

[61] Ribot EM, Fair MA, Gautom R, Cameron DN, Hunter SB, Swaminathan B, Barrett TJ (2006). Standardization of pulsed-field gel electrophoresis protocols for the subtyping of *Escherichia coli* O157: H7, *Salmonella,* and *Shigella* for PulseNet. Foodborne Pathog Dis.

[62] Barbieri NL, de Oliveira AL, Tejkowski TM, Pavanelo DB, Matter LB, Pinheiro SR, Vaz TM, Nolan LK, Logue CM, de Brito BG, Horn F (2015). Molecular characterization and clonal relationships among *Escherichia coli* strains isolated from broiler chickens with colisepticemia. Foodborne Pathog Dis.

[63] McLellan SL, Daniels AD, Salmore AK (2003). Genetic characterization of *Escherichia coli* populations from host sources of fecal pollution by using DNA fingerprinting. Appl Environ Microbiol.

[64] Johnson TJ, Wannemuehler YM, Johnson SJ, Logue CM, White DG, Doetkott C, Nolan LK (2007). Plasmid replicon typing of commensal and pathogenic *Escherichia coli* isolates. Appl Environ Microbiol.

[65] Mnif B, Harhour H, Jdidi J, Mahjoubi F, Genel N, Arlet G, Hammami A (2013). Molecular epidemiology of extended-spectrum beta-lactamase-producing *Escherichia coli* in Tunisia and characterization of their virulence factors and plasmid addiction systems. BMC Microbiol.

[66] WHO [World Health Organization] (2017). Critically important antimicrobials for human medicine – 5th rev.

