# Supplementary figures and images for: Pathogenic potential and the role of clones and plasmids in beta-lactamase-producing E. coli from chicken faeces in Vietnam
Source: BMC Vet Res. 2019 Apr 4;15:106. doi: 10.1186/s12917-019-1849-1 (PMC6449924; doi:10.1186/s12917-019-1849-1)

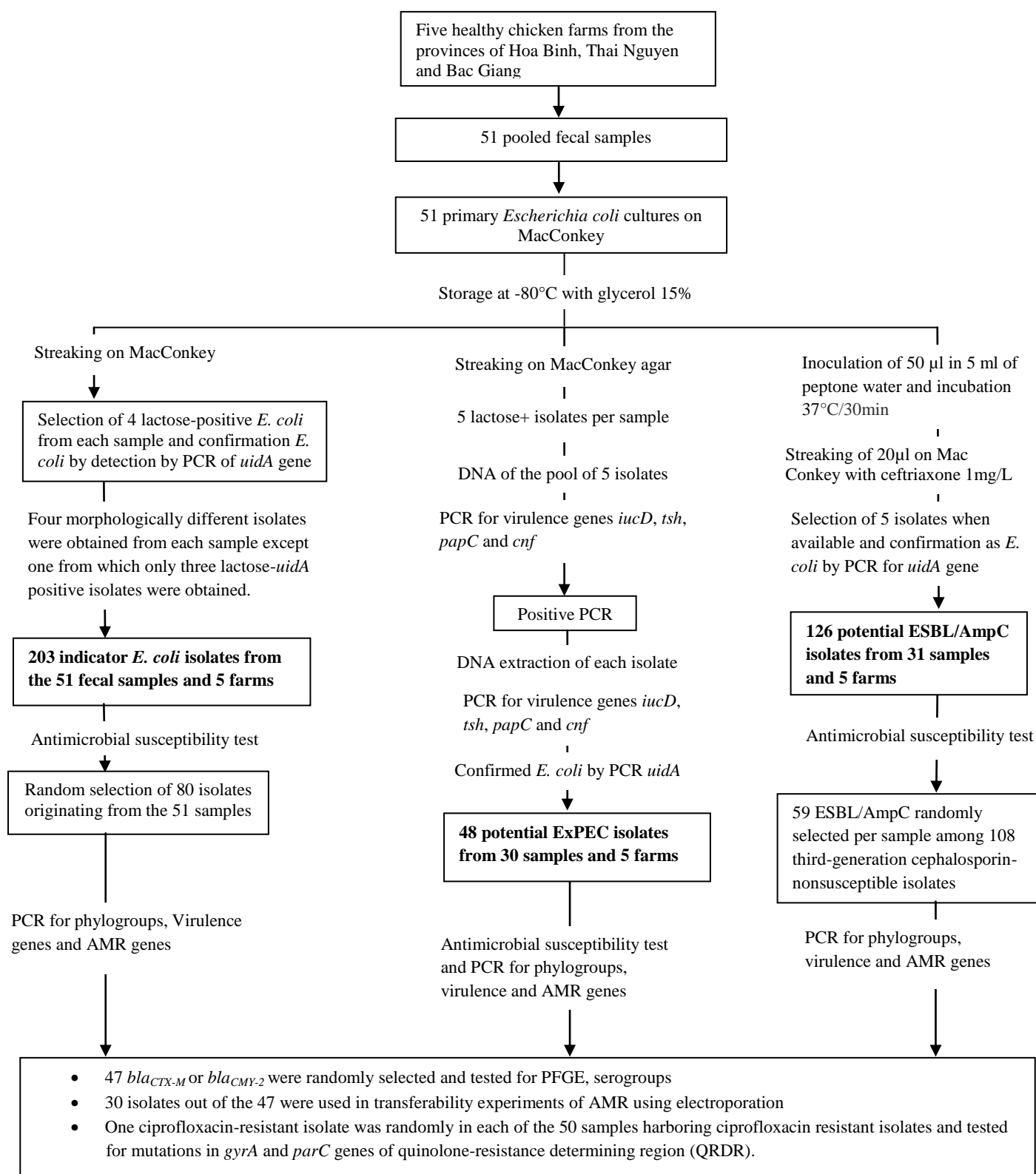

Supplement: Supplementary file 1 — Figure S1. Methodological approach used in this study. (PDF 208 kb) [file 12917_2019_1849_MOESM1_ESM.pdf]
